# Breast implant-associated anaplastic large-cell lymphoma: first case detected in a Japanese breast cancer patient

**DOI:** 10.1007/s12282-020-01064-5

**Published:** 2020-02-24

**Authors:** Yoko Ohishi, Aki Mitsuda, Kozue Ejima, Hidetomo Morizono, Tomoyuki Yano, Masahiro Yokoyama, Kengo Takeuchi, Mutsunori Fujiwara, Tetsuo Nemoto, Toshiharu Minabe

**Affiliations:** 1 Department of Breast Surgery, Nissan Tamagawa Hospital, 4-8-1, Seta, Setagaya-ku, Tokyo, 158-0095 Japan; 2 Department of Surgical Pathology, School of Medicine, Toho University, Tokyo, Japan; 3 Department of Breast Surgical Oncology, Cancer Institute Hospital, Japanese Foundation for Cancer Research, Tokyo, Japan; 4 Department of Plastic and Reconstructive Surgery, Cancer Institute Hospital, Japanese Foundation for Cancer Research, Tokyo, Japan; 5 Department of Hematology Oncology, Cancer Institute Hospital, Japanese Foundation for Cancer Research, Tokyo, Japan; 6 Division of Pathology and Pathology Project for Molecular Targets and Clinical Pathology Center, Cancer Institute Hospital, Japanese Foundation for Cancer Research, Tokyo, Japan; 7 Department of Pathology, Nissan Tamagawa Hospital, Tokyo, Japan; 8 Department of Diagnostic Pathology, School of Medicine, Showa University, Yokohama Northern Hospital, Kanagawa, Japan; 9 Department of Plastic Surgery, Saitama Medical Center, Saitama Medical University, Saitama, Japan

**Keywords:** BIA-ALCL, Breast implant, Textured surface, Anaplastic large-cell lymphoma, Axillary lymph-node swelling, Contralateral, Japan

## Abstract

This paper details the first breast implant-associated anaplastic large-cell lymphoma (BIA-ALCL) case detected in Japan. The patient, a 67-year-old Japanese woman, was diagnosed with left unilateral breast cancer 17 years ago. Induration and redness presented in the left breast, which had undergone immediate breast reconstructive surgery using a tissue expander, later replaced by a silicone breast implant (SBI). Breast ultrasound showed fluid collection around the SBI. Surgery was performed to remove the left breast implant and the fragmented capsule surrounding the implant. Postoperative pathological findings did not indicate malignancy. Nine months later, a contralateral axillary lymphadenopathy was observed, and an excisional biopsy of the axillary lymph node was performed. The patient was diagnosed with BIA-ALCL and successfully underwent adjuvant CHOP (cyclophosphamide, doxorubicin, vincristine, and prednisolone) chemotherapy.

## Introduction

Breast implant-associated anaplastic large-cell lymphoma (BIA-ALCL) was first reported by Keech and Creech in 1997 [1] and is classified as T/NK-cell non-Hodgkin’s lymphoma. Many cases of BIA-ALCL are found with late-onset seromas or masses. Pathological findings show large neoplastic cells with abundant cytoplasm and pleomorphic nuclei. BIA-ALCL may be diagnosed if CD30 is positive and anaplastic lymphoma kinase (ALK) protein is negative [2]. In many cases, BIA-ALCL is recognized within 0.8–27 years (9.75 years on average) after breast reconstruction surgery with a silicone breast implant (SBI). The onset risk is said to be latent in one of every 2,207–86,029 patients who underwent textured implant insertion [3]. Detecting BIA-ALCL, removing the SBI, and performing complete capsulectomy in the early stages reportedly lead to a good prognosis [4]. In this paper, the first Japanese breast cancer case of BIA-ALCL presenting with distant metastasis in the contralateral axillary lymph node is reported.

## Case report

**Fig. 1:**
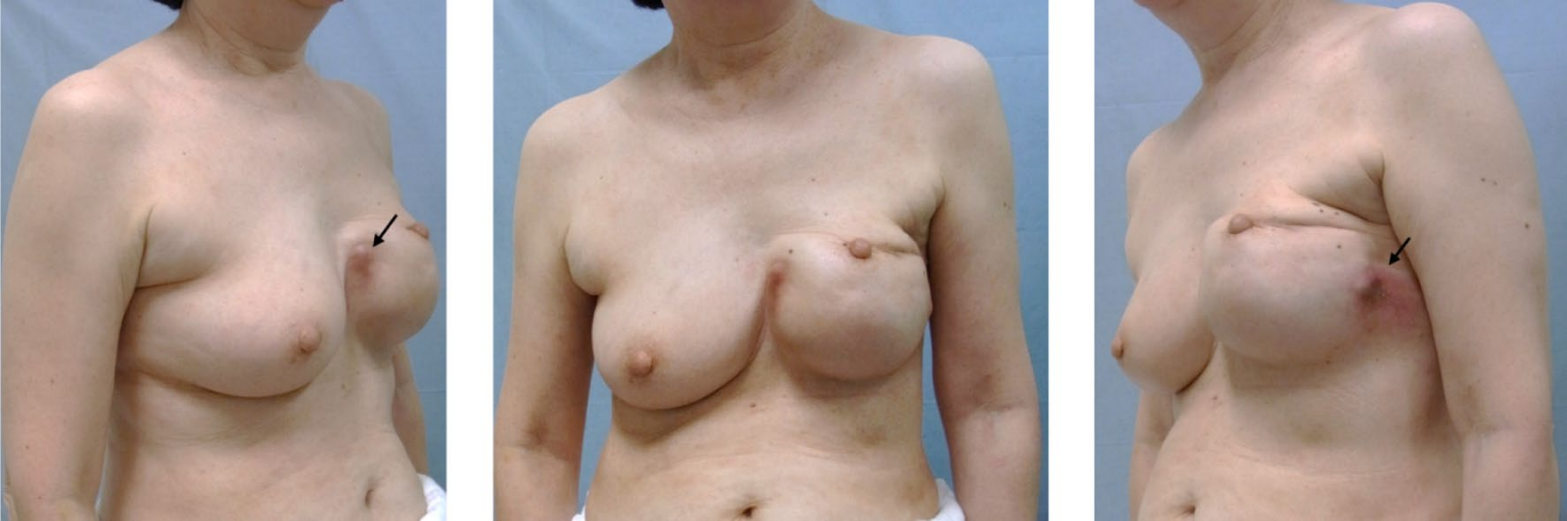
Induration with redness was observed at 4 o’clock and 10 o’clock in the reconstructed breast

**Fig. 2:**
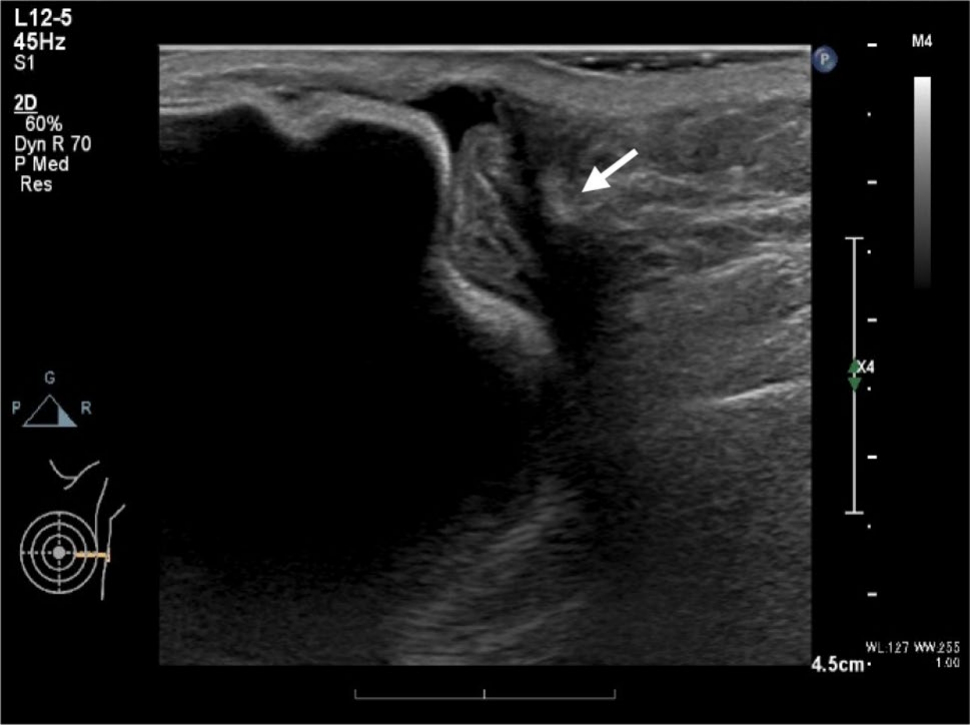
Ultrasound demonstrates fluid collection and suspended solids around the SBI with rippled shell

**Fig. 3:**
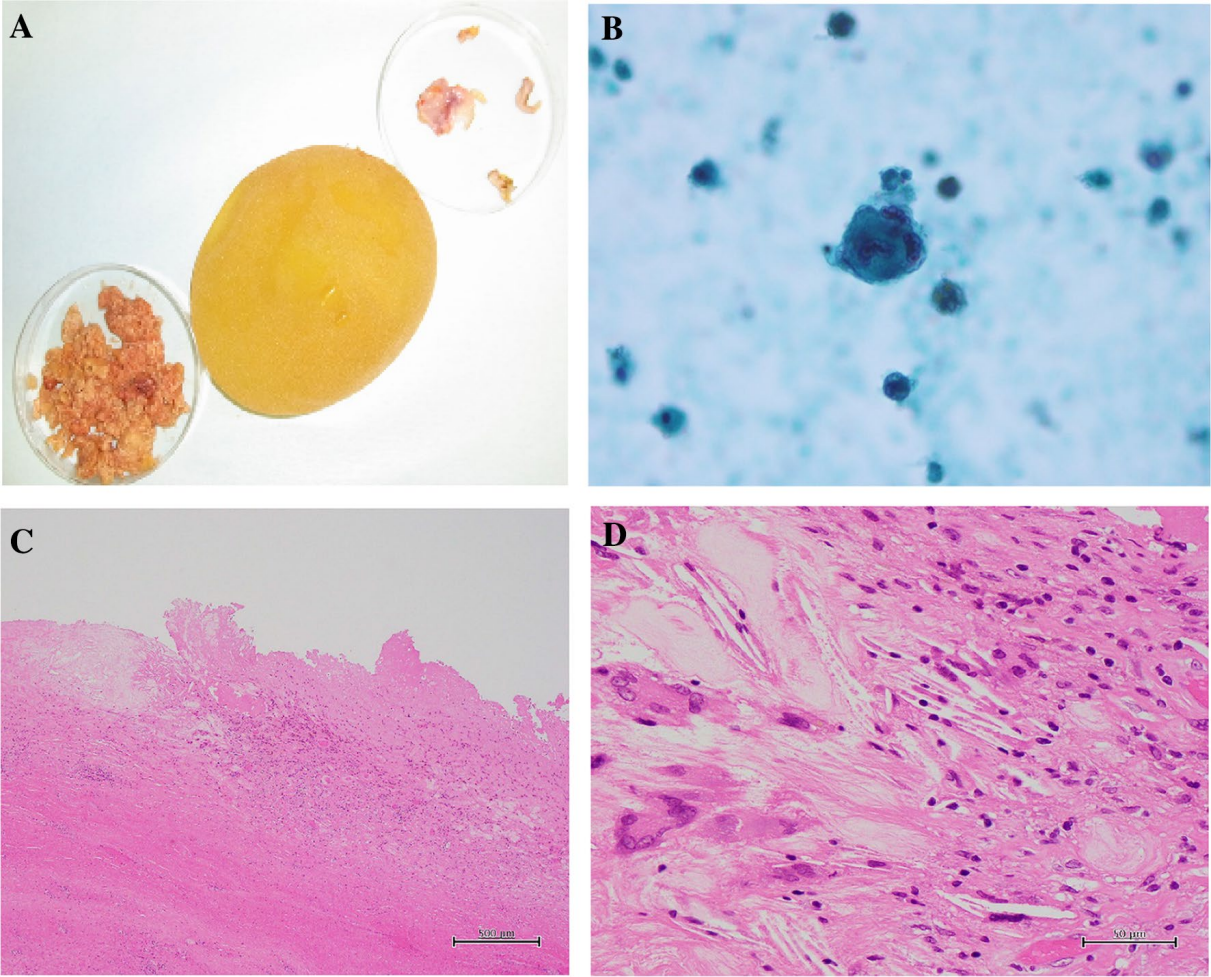
Intraoperative findings. No damage was observed to the SBI (**a**, center). The contents of the dish in the upper right are the capsule surrounding the SBI. The lower left dish contains the yellow and serous discharge with scrambled egg-like floating matters observed within the capsule. Cytological examination of the intraoperative fluid showed a small cluster of atypical cells with large, pleomorphic, hyperchromatic, and severely irregular nuclei. Malignant lesions were suspected due to clear enlargement of the nucleolus and the uneven distribution of the nuclei. The cytological finding was Class IIIb (**b**). **c,d** Fragmented capsule. The hematoxylin and eosin staining finding showed scattered chronic inflammatory cells in the necrotic area near the capsule (**c**). Scattered atypical and hyperchromatic macrophages (**d**)

A 50-year-old woman diagnosed with left unilateral breast cancer underwent mastectomy, axillary lymph-node dissection, and tissue expander insertion (model unknown) in 2002. Subsequently, the tissue expander was replaced with a textured surface SBI (McGhan Limited/410LM 220g/REF 27-LM115-220/LOT 161276). In the process of caring for her elderly parent for several years postoperatively, the patient used her pectoralis major muscles frequently. As a consequence, “breast stiffness” worsened, and prompted the patient to frequently massage the reconstructed breast. Redness on the reconstructed breast was recognized in December 2017, 15 years postoperative, but was left unattended. Symptoms worsened and she came to the hospital in June 2018. She presented with balloon-like swelling of left breast, upward shifting of the nipple and ipsilateral shoulder, and reddened and indurated areas on the medial and lateral side of the breast (Fig. 1). Ultrasound showed fluid collection around the SBI, also presenting with rippled shell (Fig. 2), and slightly enlarged bilateral axillary lymph nodes (data not shown). Because fluid collection was small, puncture was not performed before surgery. Blood tests revealed WBC 6100/μl and CRP 0.22 mg/dl. A damaged or infected breast implant was suspected, as well as the possibility of BIA-ALCL, and the SBI was removed along with as much surrounding tissue (capsule) as possible. No damage to the SBI was found, but fragmented capsules were observed during the surgical operation. As shown in Fig. 3a, yellow and serous discharge with scrambled egg-like floating matter was observed in the capsular tissue. Cytological examination of the intraoperative fluid showed a small cluster of atypical cells with large, pleomorphic, hyperchromatic, and severely irregular nuclei. Malignant lesions were suspected due to the clear enlargement of the nucleolus and the uneven distribution of the nuclei. The cytological finding was Class IIIb (Fig. 3b). Moderate nuclear atypia was recognized in large lymphoid cells with degeneration of the capsule and tissues surrounding the SBI. Fragmented capsules showed scattered chronic inflammatory cells in the necrotic area near the capsule (Fig. 3c). Atypical and hyperchromatic macrophages were seen (Fig. 3d). Results of immunohistochemistry (IHC) staining revealed CD68 (+), vimentin (+), and CK7 (−), and cells were determined to be histiocytes. Therefore, neither CD30 nor ALK was not performed. Bacterial cultures from fluid collection were negative. The postoperative diagnosis was considered to be sterile inflammation, but the possibility of BIA-ALCL could not be denied. Three months after the removal operation on the left breast, the contralateral axillary lymphadenopathy began to grow larger, and core needle biopsy was performed. In the histopathological diagnosis, most of the needle biopsy samples showed non-neoplastic changes. In a small number of regions, however, focal atypical CD30-positive cells were observed. The results of blood tests at 3 months after the removal operation showed WBC 6500/μl (neutrophil 51.4%, eosinophil 8.3%, and basophil 1.3%), CEA 0.8 ng/ml, CA15-3 9.2 U/ml, NCC-ST-439 < 1.0 U/ml, and soluble interleukin-2 receptor 477 U/ml.

**Fig. 4:**
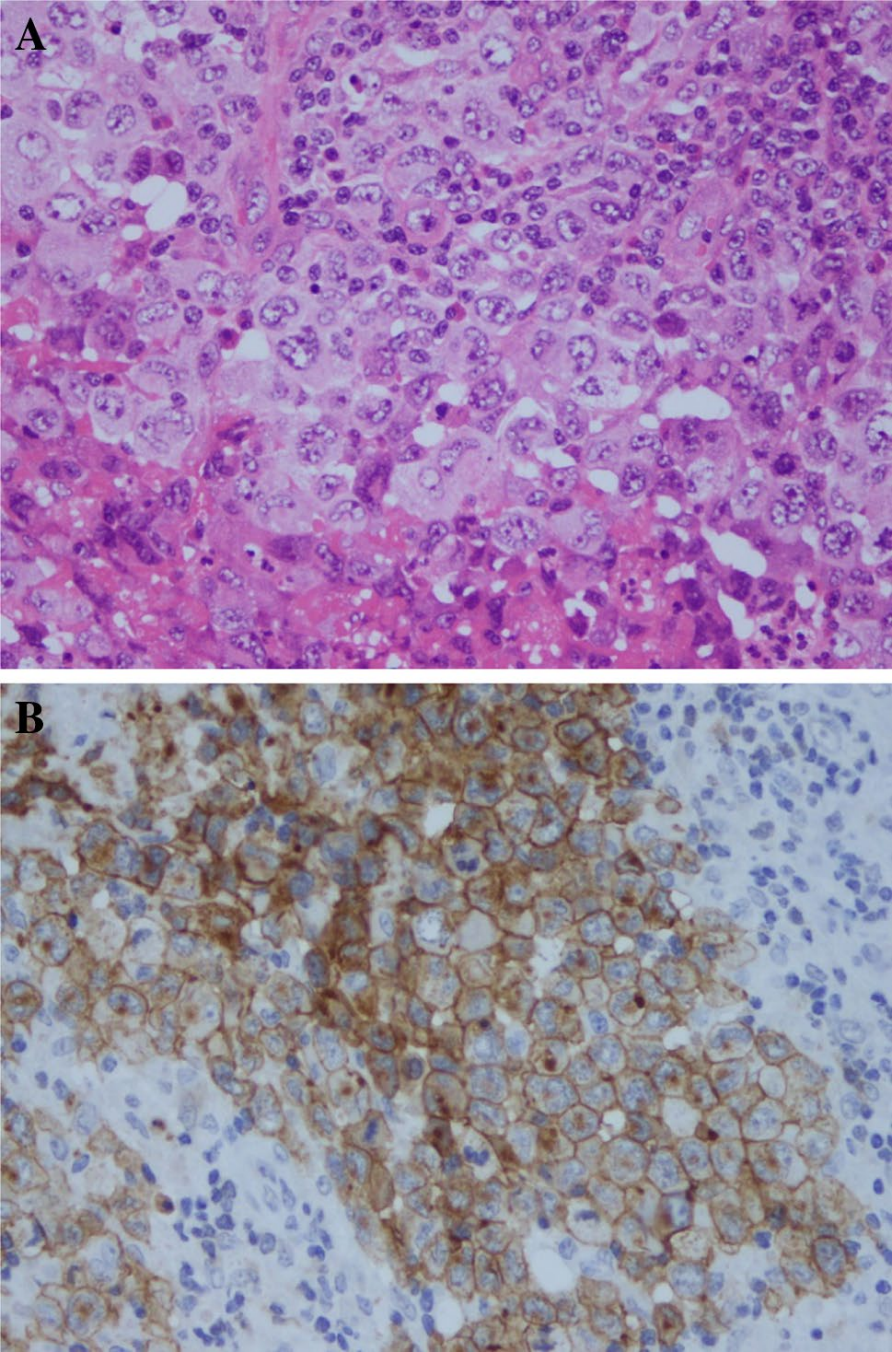
Excisional biopsy on the contralateral axillary lymph node. **a** and **b** were photographed at 400× original magnification. The hematoxylin and eosin staining finding showed a large atypical lymphoid cell proliferation with a pleomorphic nucleus (**a**). The neoplastic cells were stained strongly by CD30 immunohistochemistry (**b**)

By 5 months postoperatively, the enlargement of the contralateral axillary lymph node had progressed even more. Now, fine needle aspiration cytology resulted in a Class IIIb diagnosis. Excisional biopsy was then performed on the contralateral axillary lymph node. The pathological findings showed proliferation of large atypical lymphoid cells with pleomorphic nuclei. (Fig. 4a). The result of IHC staining revealed CD30 (+), ALK (−), CD4 (weakly positive), CD8 (−), CD3 (−), CD20 (−), CD56 (−), GranzymeB (+), AE1/3 (−), EMA (−), and CK5/6 (−).The patient was diagnosed with BIA-ALCL due to CD30 positivity (Fig. 4b) and ALK negativity. The patient was in Stage IV and was transferred to a specialized institution to receive adjuvant CHOP (cyclophosphamide, doxorubicin, vincristine, and prednisolone) chemotherapy every 21 days for 6 cycles. Seven months have passed, since the excisional biopsy was performed, and a complete metabolic response was confirmed by PET-CT.

## Discussion

The widespread use of silicone implants in breast reconstruction started in the 1960s [5]. In Japan, SBIs were not covered by insurance until July 2013, when the national health insurance system allowed implants for breast reconstruction. However, the Japan Oncoplastic Breast Surgery Society (JOPBS) allows breast reconstruction surgery to be performed only by physicians and facilities certified by the Society [6]. In Japan, only Allergan breast implants/tissue expander are approved involving “Biocell”: macro-texture. According to the 2018 annual report of the JOPBS, 6,582 SBIs were used in that year [7]. In contrast, the 2018 Plastic Surgery Statistics Report issued by the American Society for Plastic Surgeons says that there were 78,814 SBIs, about 12 times more than the figure in Japan, used that year. By ethnicity, Caucasians accounted for 71% of the women who had reconstructive surgery, African-Americans 13%, Asian/Pacific Islanders 4%, Hispanics 11%, and others 1%. Asians use SBIs less frequently [8]. According to Average breast size worldwide on WorldData.info, the most common cup size of women is A (54–304 ml, 179 ml on average) in Japan in marked contrast with F or greater (645–2986 ml, 1668 ml on average) in the United States (Caucasians) and C (147–831 ml, 489 ml on average) in Spain [9, 10]. By race, Caucasians account for 92.1% of BIA-ALCL patients, according to the patient registry and outcomes for breast implants and anaplastic large-cell lymphoma etiology and epidemiology (PROFILE) [11]. Furthermore, among major lymphoma subtypes, ALCL-ALK negative was 7.8% in North America and 2.6% in Asia [12]. The findings suggested that certain ethnic backgrounds and also breast size and the number of SBIs used may be associated with the incidence of BIA-ALCL.

Additionally, bacterial biofilm is one possible cause of BIA-ALCL [13]. Reportedly, BIA-ALCL may easily occur in patients undergoing textured implants [14, 15]. These types of implants do not require postoperative massage, creating a great advantage over smooth implants. However, in this case, the patient frequently massaged her breast reconstructed with a macro-textured implant. We hypothesize that the frequent massage may have been the cause of “latent effusion” and “inflammatory rash” along with persistent chronic inflammation. In addition, a differential diagnosis of the symptoms recognized in this case, namely seroma, redness, and induration of the breast, requires consideration of the possibility of local recurrence as well as BIA-ALCL.

The development of BIA-ALCL is also associated with persistent chronic inflammation [16]. Chronic inflammation may induce B-cell-derived lymphomas such as MALT (mucosa-associated lymphoid tissue) lymphoma and pyothorax-associated lymphoma (PAL) induced by Helicobacter pylori [17] and Epstein–Barr virus [18], respectively. Comprehensive next-generation sequence analysis of BIA-ALCL cases revealed sequence variants that activate JAK/STAT signaling. High copy-number amplification of TNFRSF11A and PDGFRA was detected, which could be expected as therapeutic targets [19]. Currently, five cases of BIA-ALCL associated with germline TP53 mutation have been reported [19–22].

BIA-ALCL may be diagnosed if a neoplasm composed of large lymphoid cells that are CD30 positive and ALK negative is found. However, CD30 can also be positive in Hodgkin’s lymphoma, Epstein–Barr virus-associated lymphomas, and non-lymphoid neoplasms. CD30 positivity in ALCL is defined as 75% or more of tumor cells expressing positive CD30. For this reason, it should be noted that being CD30 positive does not always equal to ALCL [23]. The pathological diagnosis was difficult in this case, because the samples collected at the time of SBI removal or axillary lymph-node needle biopsy contained only a few tumor components with degeneration. Primary lesion showed widely necrotic tendency. Histologically, there was no solid collection of the living lymphoma cells near the capsule. Therefore, it would be difficult to diagnose BIA-ALCL even if the capsule could be excised in en bloc at the first operation. Excisional biopsy of the axillary lymph node allowed for the collection of more tissue and made the correct diagnosis possible.

In hematoxylin and eosin staining, ALCL may morphologically look like a poorly differentiated carcinoma. As this patient had a history of breast cancer, our first impression of the H&E specimen suspected of axillary lymph-node metastasis from poorly differentiated adenocarcinoma. However, the patient had undergone breast reconstruction with the SBI, and BIA-ALCL needed to be included in the differential diagnosis. Compared to BIA-ALCL after breast augmentation, BIA-ALCL after breast cancer reconstruction appears to be more difficult to diagnose, because it requires differentiation from cancer recurrence.

According to the Food and Drug Administration (FDA) Safety Communication, 573 BIA-ALCL patients were analyzed as of July 6, 2019, and 33 of these patients were reported to have died. Of the 33 died patients, the manufacturer was identified in 13, of which 12 used Allergan breast implants. This led the FDA to request Allergan to voluntarily recall the implants and tissue expanders in question from the global market (as written in an FDA release dated July 24, 2019) [24]. We should take into account the fact that the environment surrounding breast surgery and reconstruction in Japan has been changing after the confirmation of the first case of BIA-ALCL in Japan.

## Conclusion

The number of BIA-ALCL cases is expected to increase in the future, and conducting lifetime surveillance postoperatively is important. Since BIA-ALCL must be detected and treated early, it may be imperative to raise awareness about the disease not only among breast surgeons, plastic surgeons, and pathologists but also among patients. Patients with a difficult diagnosis of BIA-ALCL at the time of SBI removal need to undergo strict follow-up. Diagnosing lymphoma is often difficult in needle biopsies performed on an axillary lymphadenopathy. Therefore, an excisional biopsy should be performed to ensure an accurate differential diagnosis of BIA-ALCL from recurrent and poorly differentiated breast adenocarcinoma.
